# Single-prolonged stress induces apoptosis in dorsal raphe nucleus in the rat model of posttraumatic stress disorder

**DOI:** 10.1186/1471-244X-12-211

**Published:** 2012-11-27

**Authors:** Dongjuan Liu, Bing Xiao, Fang Han, Enhua Wang, Yuxiu Shi

**Affiliations:** 1 Department of Electron Microscopy, Basic Medical Sciences College, China Medical University, 92 North 2nd Road, Shenyang 110001, Liaoning, China; 2 Department of Histology and Embryology, Basic Medical Sciences College, China Medical University, Shenyang, China; 3 Institute of pathology and pathophysiology, China Medical University, Shenyang, China

**Keywords:** Posttraumatic stress disorder, Dorsal raphe nucleus, Neuron, Apoptosis, Thiamine monophosphatase, Cytochrome c

## Abstract

**Introduction:**

Post-traumatic stress disorder (PTSD) is an anxiety disorder that develops after exposure to a life-threatening traumatic experience. Meta-analyses of the brainstem showed that midsagittal area of the pons was significantly reduced in patients with PTSD, suggesting a potential apoptosis in dorsal raphe nucleus after single-prolonged stress (SPS). The aim of this study is to investigate whether SPS induces apoptosis in dorsal raphe nucleus in PTSD rats, which may be a possible mechanism of reduced volume of pons and density of gray matter.

**Methods:**

In this study, rats were randomly divided into 1d, 7d and 14d groups after SPS along with the control group. The apoptosis rate was determined using annexin V-FITC/PI double-labeled flow cytometry (FCM). Levels of Cytochrome c (Cyt-C) was examined by Western blotting. Expression of Cyt-C on mitochondria in the dorsal raphe nucleus neuron was determined by enzymohistochemistry under transmission electron microscopy (TEM). The change of thiamine monophosphatase (TMP) levels was assessed by enzymohistochemistry under light microscope and TEM. Morphological changes of the ultrastructure of the dorsal raphe nucleus neuron were determined by TEM.

**Results:**

Apoptotic morphological alterations were observed in dorsal raphe nucleus neuron for all SPS-stimulate groups of rats. The apoptosis rates were significantly increased in dorsal raphe nucleus neuron of SPS rats, along with increased release of cytochrome c from the mitochondria into the cytoplasm, increased expression of Cyt-C and TMP levels in the cytoplasm, which reached to the peak of increase 7 days of SPS.

**Conclusions:**

The results indicate that SPS induced Cyt-C released from mitochondria into cytosol and apoptosis in dorsal raphe nucleus neuron of rats. Increased TMP in cytoplasm facilitated the clearance of apoptotic cells. We propose that this presents one of the mechanisms that lead to reduced volume of pons and gray matter associated with PTSD.

## Background

Post-traumatic stress disorder (PTSD) is a psychiatric condition that develops after being exposed to a life-threatening traumatic experience. It is characterized with long-term symptoms of continuous experiences of the traumatic event, avoidance of stimuli associated with the trauma, numbing of general responsiveness, and increased arousal [1-3]. The pathophysiology of PTSD has been widely studied [4]. One of the core neuroendocrine abnormalities related to the disorder is the dysfunction of the hypothalamic-pituitary-adrenal (HPA) axis, characterized by low levels of adrenocorticotropic hormone (ACTH), plasma cortical and urinary cortisol and enhanced suppression of cortisol in response to low-dose dexamethasone administration [5,6]. Volumetric imaging studies have shown abnormal brain morphology in PTSD when compared with control subjects, and volumetric image analysis of the brainstem showed smaller gray matter volume in the pons with PTSD [7]. However, the mechanism underline this finding has not been elucidated.

The dorsal raphe nucleus is located on the midline of the brainstem. It is a part of the raphe nucleus, locate at upper part of pons to caudal subdivisions of the oculomotor nucleus [8]. Dorsal raphe nucleus involves in multiple behavioral and physiological processes [9], and plays an important role in response to traumatic stimuli. In our previous study, we have reported that there is an increase of 5-HT1A in the dorsal raphe nucleus in the rats upon Single-prolonged stress (SPS) [10], which might be related to apoptosis in the dorsal raphe nucleus.

Apoptosis is a genetically programmed, morphologically distinct form of cell death that can be triggered by a variety of physiological and pathological stimuli [11]. There are distinct mechanisms that execute apoptosis according to various apoptotic stimuli. Apoptosis can be induced by two major pathways: the intrinsic pathway (mitochondria-dependent pathway) and the extrinsic pathway (death receptor-dependent pathway). Both of the pathways are regulated by caspases [12]. Caspases transduce the apoptotic signal cascade and engage the cellular targets that lead to programmed cell death [13,14]. In response to the apoptotic stimuli, mitochondria were impaired, resulting in the release of cytochorme C ( Cyt-C) from mitochondria into cytoplasm [15]. Cyt-C, a key enzyme in the respiratory chain, not only takes part in regulation of apoptosis but also is the key in mediating apoptosis upon being released into the cytosol. Cyt-C is also an intermediate in apoptosis, a controlled form of cell death used to kill cells in the process of development or in response to infection or DNA damage [16]. Thiamine monophosphatase (TMP), the hallmark of lysosomal functional state, is involved in the process of cell degeneration, necrosis and clearance of apoptotic cells [17,18]. SPS, which shows time-dependent dysregulation in the HPA axis, is one of the widely used animal models for PTSD research [19].

In this study, in effort to elucidate the mechanisms underlying decreased volume of pons associated with PTSD, we employed the SPS model to investigate the changes of apoptosis rate, Cyt-C and TMP in dorsal raphe nucleus neurons as well as the morphological changes in ultrastructure of the dorsal raphe nucleus neuron by PTSD. Our data support the hypothesis that SPS-induced apoptosis in dorsal raphe nucleus contributes to the reduced volume of pons and density of gray matter in patients with PTSD.

## Materials and methods

### Experimental animals

Eight weeks-old Wistar rats (approximately 180 -200 g), provided by the Animal Experimental Center of China Medical University, were used for all experiments. The rats were housed singly in clear polycarbonate cages (46 × 24 × 20 cm) for one week prior to the experiments. All rats were habituated to their cage and given standard food pellets and water. They were housed under a reversed the 12 h:12 h light/ dark cycle (lights off at 10.00 a.m) and ambient temperature (23 ± 2°C) with humidity of 55 ± 5%. Rats were exposed to SPS on the day when weighed approximately between 230 and 250 g. All animal protocols were carried out in accordance with the Guidance Suggestions for the Care and Use of Laboratory Animals formulated by the Ministry of Science and Technology of the People’s Republic of China [20] and approved by Welfare & Ethics Committee of Exprimental, China Medical University. All efforts were made to reduce the number of animals used and to minimize animal pain during the experiment.

### Model establishment and grouping

After lab adaptation and handling, the rats were randomly assigned to one of the four groups and each group contains twenty rats. One group was served as a control group, while the others were SPS groups. The control rats were killed after the acclimation period, while the SPS groups rats were exposed to SPS procedure. The SPS model was prepared according to the previous report [21,22]. Briefly, each rat was restrained for two hours by placing it inside a clear disposable plastic bag with only the tail protruding. The plastic bag was closed with tape at the base of the tail. The size of plastic bags was adjusted according to the size of the rat in order to achieve complete immobilization. A hole in the plastic bags allowed the rats to breathe freely. After immobilization, rats were individually placed in a clear acrylic cylinder (240 mm in diameter and 500 mm height), and two thirds of the cylinder from the bottom was filled with water (24°C). The rats were forced to swim for 20 min. Following 15 min of recuperation, rats were kept undisturbed in their home cage. Consistent with time-dependent sensitization, behavioral experiments are generally undertaken 1 ~ 14 days after the SPS procedure [23]. In this study, the SPS groups of animals were randomly assigned into three different groups depending on the days after SPS-stimulus: the group of 1 day after the SPS procedure (n = 20; SPS 1d group), the group of 7 days after the SPS procedure (n = 20; SPS 7d group),and the group of 14 days after the SPS procedure (n = 20; SPS 14d group). Each group included 20 rats, in which five rats were used for frozen sections, five for Western Blotting, five for flow cytometry, and five for TEM.

### Perfusion-based sections

The rats of each group were anesthetized with 10% chloral hydrate. The hearts were exposed, and the left ventricles were perfused with 200–300 mL of 0.9% saline via a catheter through the ascending aorta until a colorless infusion was achieved, followed by perfusion with a 300 mL of 4% paraformaldehyde (PFA) [24]. The whole brain were rapidly removed and dissected on ice, followed by 6–10 h of post-fixation in 4% PFA at 4°C. After being immersed in 20% sucrose solution and frozen in liquid nitrogen, coronal sections of the brain tissue were cut into slices of 12 μm in thickness and stored at −20°C for enzymohistochemistry morphological studies.

### Enzymohistochemistry analysis on expression of Cyt-C using TEM

Sections of brain tissue in each group were washed with 0.1 M phosphate-buffered saline (PBS) ( pH 7.4) for 30 min at room temperature, and incubated with incubation buffer containing (Cyt-C 10 mg, catalase 1 mg, 0.2 M PBS 5 ml, DAB 10 mg, distilled water 5 ml ) at 4°C overnight. The samples were washed three times with PBS after incubation. To assess nonspecific staining, a few sections in every experimental group were incubated in PBS without incubation buffer. After washing, the samples were fixed with 1% osmium tetroxide at 4°C for 20 min and washed in PBS several times, dehydrated in gradient series (20–100%) of ethanol and then in (100%) acetone, infiltrated with Epon 812, and finally polymerized in pure Epon 812 at 65°C for 48 h. The location of the dorsal raphe was determined on semi-thin sections. Ultra-thin sections (70 nm) were cut with ultramicrotome, collected on copper grids, and stained with 4% uranyl acetate. Five sections were selected from each group. Ten visual fields (about 250 cells) of the dorsal raphe nucleus in each section were examined with TEM (JEM-1200EX, Japan). The morphological changes and expression of Cyt-C of mitochondria were determined. The rates of change were calculated with the following formula: rate = (numbers of mitochondria with morphological changes /total numbers of mitochondria in the cell) × 100%.

### Western blotting analysis of Cyt-C expression

Rats were anesthetized with 10% chloral hydrate and decapitated. The brain tissues were quickly removed and placed on ice. The dorsal raphe nucleus fragments were dissected from the brain tissues under a stereomicroscope in each experimental group according to the atlas of rats [25]. Proteins were extracted from the dorsal raphe nucleus samples of normal control rats or SPS rats through homogenization, ultrasonic dispersion and centrifugation. Extracted proteins were resuspended in the samples buffer containing 200 mM tris-buffered saline (TBS*)*, pH 7.5, 4% sodium dodecyl sulfate (SDS*)*, 20% glycerol, 10% β- mercaptoethanol and boiled for 3 min. The protein fraction (50 μg/lane) prepared from each group was separated by 12% (w/v) gradient sodium dodecyl sulfate (SDS)-polyacrylamide gel electrophoresis (PAGE) and transferred to a polyvinylidene fluoride (PVDF) membrane (Millipore, Bedford, MA, USA) using a semi-dry blotting apparatus (Bio-Rad Laboratories, Inc. Hercules, CA, USA). The membrane was blocked with 5% dried skim milk, 0.05% Tween-20 in TBS at room temperature for 2 h and incubated with rabbit polyclonal antibody against Cyt-C IgG (Bipec Biopharma Corporation, U.S.A. 1:200) at 4°C for 2 h. The membranes were washed three times with TBST (tris buffer with saline and tween 20), and then incubated with the anti-mouse IgG-HRP (Santa Cruz, U.S.A.; 1:5000) for 2 h at room temperature followed with TBST before visualization using enhanced chemiluminescence (ECL; Amersham Pharmacia Biotech, Buckinghamshire, UK). To confirm equal protein loading, the same blots were re-incubated with antibodies specific for β-actin (Abcam, British; 1:1,000) and detected with the ECL. The Optical Density (OD) was analyzed with the Gel Image Analysis System.

### Flow cytometry analysis of cell apoptosis rate in dorsal raphe nucleus

Rats were anesthetized with 10% chloral hydrate and decapitated. The brain tissues were quickly removed and placed on ice. The dorsal raphe nucleus fragments were dissected from the brain tissues under a stereomicroscope in each experimental group according to the atlas of rats [25]. Single cell suspensions were then generated in cold PBS buffer, and the final concentrations were adjusted to 1 × 10^4^/ml. 1 ml of suspensions were centrifuged it at 1000 rpm for 10 min at 4°C and washed with 1 ml cold PBS three times. Cell pellets were suspended in 200 μl of binding buffer, and then incubated with 10 μl of Annexin V-FITC and 5 μl of Propidium Iodide at room temperature for 15 min. Finally, 300 μl of binding buffer was added into the incubation solution and was analyzed by flow cytometry (Becton Dicklnson, USA) in 1 h. About 10^4 ^of cells were analyzed by flow cytometry for each group. The apoptosis rate was calculated with the following formula: (number of apoptotic cells /total cells) × 100%.

### Assessing the morphological change in dorsal raphe nucleus using TEM

Dissected dorsal raphe nucleus samples were separated into 1 mm^3 ^pieces, fixed with 2.5% glutaraldehyde at 4°C. The pieces were post-fixed in 1% osmium tetroxide for 2 h at 4°C, rinsed in 0.1 M PBS (pH 7.4) several times, dehydrated in a gradient series (20–100%) of ethanol and then in 100% acetone, infiltrated with Epon 812, and finally polymerized in pure Epon 812 at 65°C for 72 h. The dorsal raphe nucleus was positioned in semi-thin sections. Ultra-thin sections (70 nm) were cut with ultramicrotome, collected on copper grids, stained with uranyl acetate and lead citrate. Five sections were selected from each group. Ten visual fields (about 250 cells) of the dorsal raphe nucleus in each section were examined with TEM (JEM-1200EX, Japan). The rates of the apoptotic cells was calculated with the following formula: rate = (number of the apoptosis cells /total cells) × 100%.

### Enzymohistochemistry analysis of TMP expression using light microscope

Brain tissue sections in each group were washed with 0.1 M phosphate-buffered saline (PBS) (pH 7.4) for 30 min at room temperature, and samples were then incubated with TMP buffer [containing 15.29 g of TMP, 0.05 M of acetic acid buffer 20 ml (pH 3.9), 35.405 mg of CeCl_3_, 2.5 g of sucrose, 10 mg of DAB, 30 ml of distilled water, 0.000075 ml of TritonX-100] for 80 min at 37°C. To assess nonspecific staining, a few sections in every experimental group were incubated in PBS without incubating buffer. The sections were washed three times with PBS after incubation. Finally, 1% Vulcanization amine was used as chromogen for 1 min until the brown color appeared. Slides were then dehydrated and mounted with neutral gum. Five sections were selected from each group. Five visual fields of the dorsal raphe nucleus in each section were selected for examination (× 40). The optical density (OD) of TMP enzymohistochemistry positive cells in each field were analyzed using the MetaMorph/DPIO/BX41 morphology image analysis system, and the average of OD were determined. The rates of the TMP positive enzymohistochemistry staining cells were calculated with the following formula: rate = (number of the TMP positive cells/total cells) × 100%.

### Enzymohistochemistry analysis of TMP expression using TEM

After staining with 1% vulcanization amine as described above, the sections were washed with PBS. Then the sections were fixed in 1% osmium tetroxide for 20 min at 4°C, rinsed in 0.1 M PBS (pH 7.4) several times, dehydrated in ethanol gradient (20–100%) and then in 100% acetone, infiltrated with Epon 812, and finally polymerized in pure Epon 812 at 65°C for 48 h. The dorsal raphe nucleus was positioned in the semi-thin sections. Ultra-thin sections (70 nm) were cut with ultramicrotome, collected on copper grids, and stained with uranyl acetate. Five sections were selected from each group. Ten visual fields (about 250 cells) of the dorsal raphe nucleus in each section were examined with TEM (JEM-1200EX, Japan). The rate of TMP-positive lysosome were calculated with the following formula: rate = (number of positive expression of lysosome /total lysosome) × 100%.

### Statistical analysis

The results were expressed as mean ± SEM. The differences between control group and the SPS groups were analyzed by one-way analysis of variance (ANOVA) using SPSS 13.0 software. In the case of significance with the ANOVA, post hoc comparisons were made using the Tukey’s test to compare between groups. A level of P < 0.05 was considered to be statistically significant.

## Results

### Enzymohistochemistry analysis of Cyt-C by TEM

**Figure 1:**
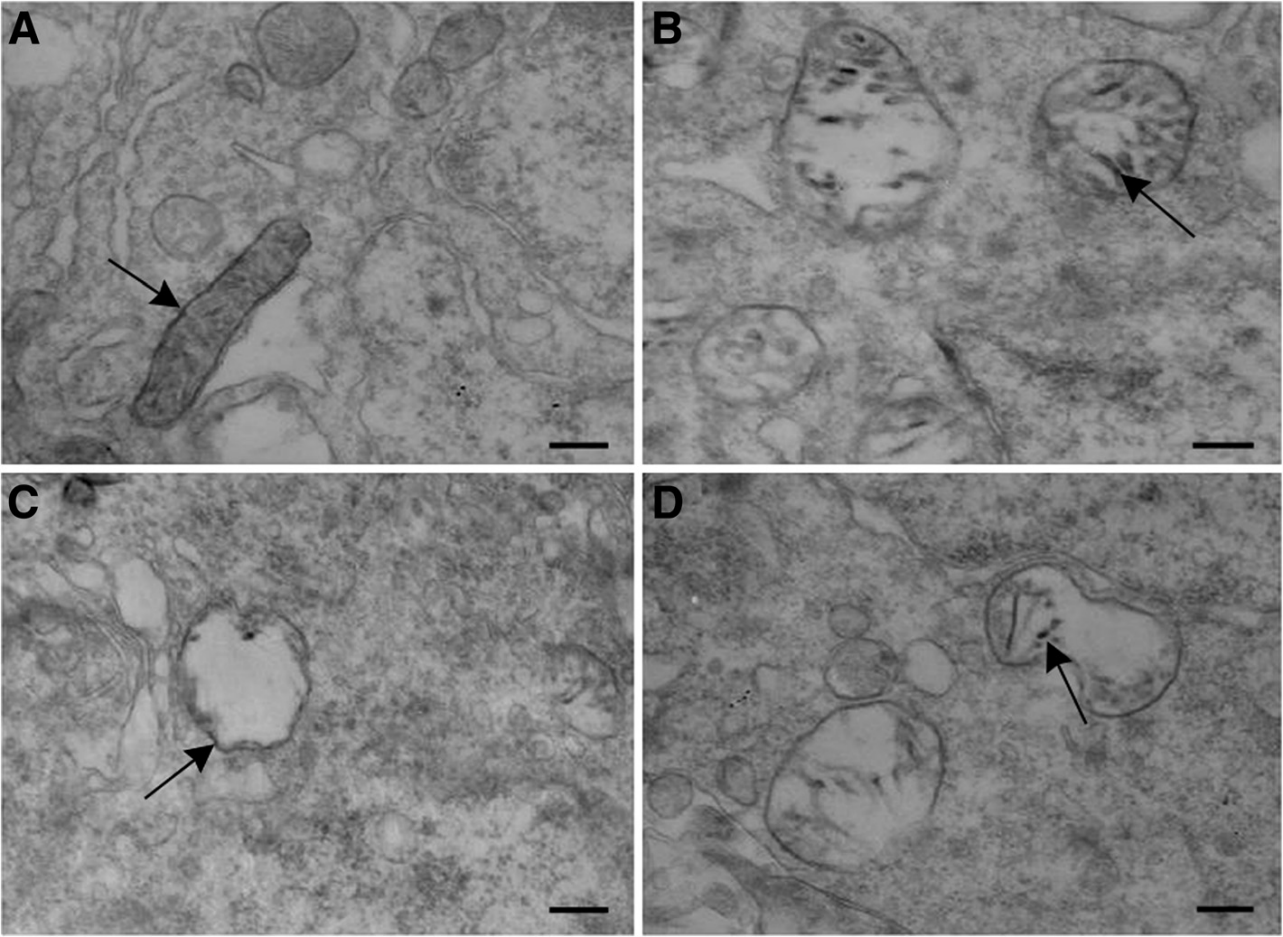
**The distribution of Cyt-C in mitochondria were found in dorsal raphe nucleus by TEM in each group (Figure**1A-D**), Positive expression of Cyt-C, high electron-dense black granule(↑) in exterior and interior membrane of mitochondria. A**: Mitochondria is intact, positive granule of Cyt-C were plentiful in control group; **B**: Part of Mitochondrial cristae fracture and membrane disruption,positive granule of Cyt-C were partly decreased in SPS1d group; **C**: Part of Mitochondrial vacuolization,positive granule of Cyt-C were obvious decreased in SPS 7d group. **D**: Part of Mitochondrial cristae fracture and membrane disruption,positive granule of Cyt-C were partly decreased in SPS14d group. All images are taken with identical magnification (Scale bar =500 nm).

**Figure 2:**
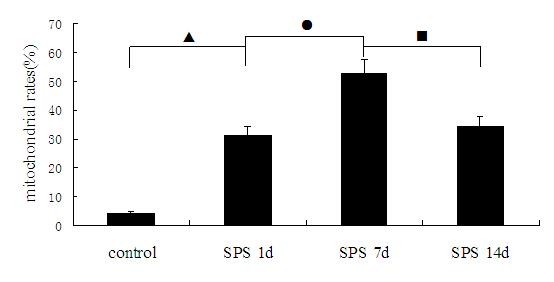
**The rates of mitochondrial morphological change were calculated by TEM at different time points in dorsal raphe nucleus (mean ± SEM).** Most change was found at SPS 7d. *P* < 0.001 SPS vs. control group. Tukey’s test: ▲*P* < 0.05 SPS 1d vs. control group. ●*P* < 0.05 SPS 7d vs. SPS 1d. ■*P* < 0.05 SPS 14d vs. SPS 7d.

To investigate whether SPS induces apoptosis in dorsal raphe nucleus, we first examined Cyt-C expression by TEM. As shown in Figure 1, Cyt-C expression were detected and presented as high electron-dense black dots, which were distributed in the exterior and interior membranes of mitochondria. In contrast to the normal mitochondrial structure in control group (Figure 1A), many abnormal mitochondria were observed in SPS groups. Mitochondrial cristae fracture, vacuolization and membrane disruption were observed in samples from the group SPS 1d (Figure 1B), SPS 7d (Figure 1C) and SPS 14d (Figure 1D). In comparison to the control group, Cyt-C expression in the exterior and interior membranes of mitochondria were partly decreased in the SPS groups, with the translocation of Cyt-C from mitochondria to the cytoplasm of the cells in the SPS groups. Furthermore, this change was most obvious at SPS 7d. The rates of mitochondrial morphological change were reported in Figure 2 (Figure 2, F = 184.741 df = 3, 20; *P* < 0.001. Tukey’s test *P* < 0.05).

### Western blotting analysis of Cyt-C

**Figure 3:**
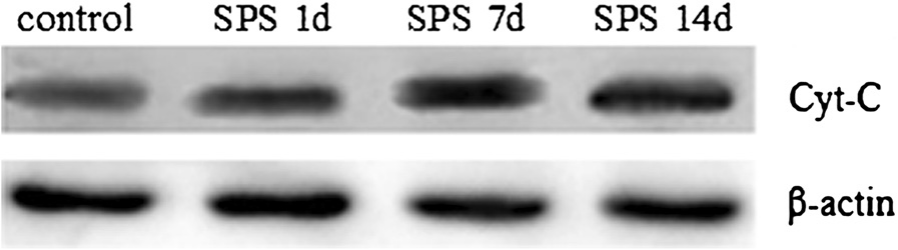
**Western Blotting showed that the protein expression of Cyt-C increased in dorsal raphe nucleus of SPS rats compared with control levels.** Protein expression of Cyt-C peaked at SPS 7d.

**Figure 4:**
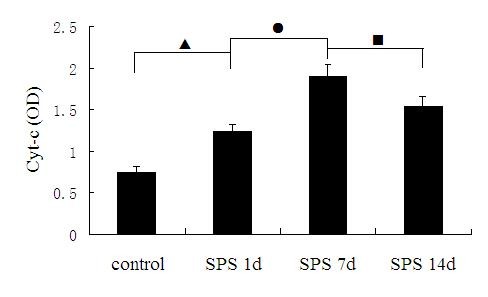
**The mean optical densities of Cyt-C expression was shown at different time points in dorsal raphe nucleus, and peaked at SPS 7d (mean ± SEM). *P*** **< 0.001 SPS vs. control group.** Tukey’s test: ▲*P* < 0.05 SPS 1d vs. control group. ●*P* < 0.05 SPS 7d vs. SPS 1d. ■*P* < 0.05 SPS 14d vs. SPS 7d.

We confirmed expression of Cyt-C by western blotting analysis. As shown in Figure 3, the Cyt-C and β-actin proteins were detected at 17 and 42 kDa, respectively, and the mean values of the band densities of the control group were set as 100%. The result showed that Cyt-C expressions in the SPS groups were significantly increased in comparision to that of the control group. The peak of increase of Cyt-C levels occurred in SPS 7d, but gradually decreased at SPS 14d. The mean optical densities of Cyt-C expression was indicated in Figure 4 (Figure 4, F = 98.829 df =3, 20 ; *P* < 0.001. Tukey’s test *P* < 0.05).

### Analysis apoptosis rate by flow cytometry

**Figure 5:**
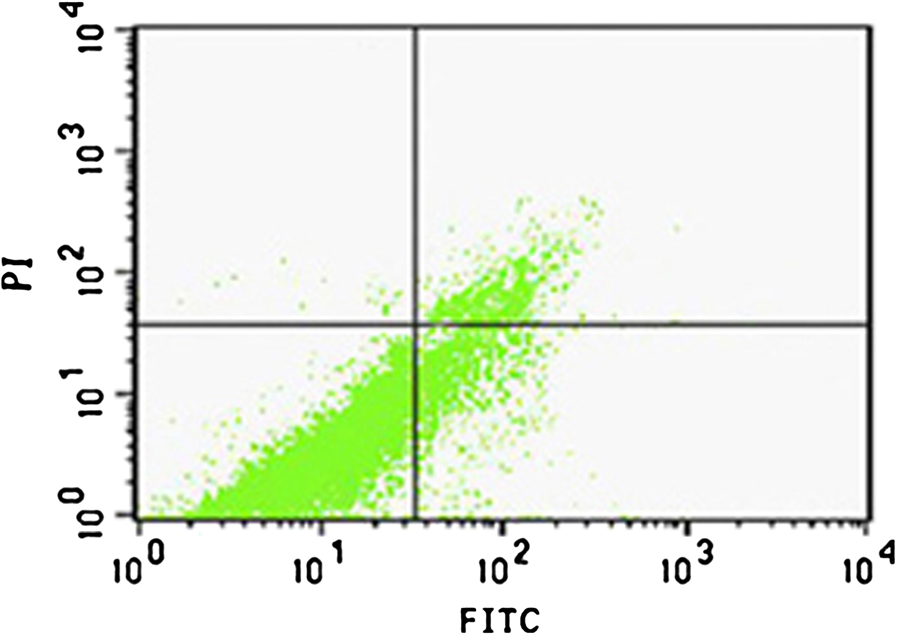
**Flow cytometry detected the apoptosis rate in dorsal raphe nucleus at SPS7d.** Flow cytometry pictographs: X-axis and Y-axis represents the relative intensity values of the fluorescence signal FITC and PI.

**Figure 6:**
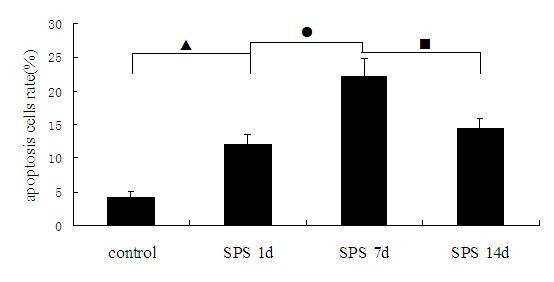
**Double-labeled flow cytometry analyzed apoptosis rate in dorsal raphe nucleus at different time points (mean ± SEM).** The apoptosis rate peaked at SPS 7d. *P* < 0.001 SPS vs. control group. Tukey’s test: ▲*P* < 0.05 SPS 1d vs. control group. ●*P* < 0.05 SPS 7d vs. SPS 1d. ■*P* < 0.05 SPS 14d vs. SPS 7d.

We further determined the numbers of apoptotic cells upon SPS by flow cytometry using Annexin V-FITC /PI staining. Rats in the SPS 7d group had considerable amounts of apoptotic cells in dorsal raphe nucleus cells (Figure 5). These cells were in cluster-shape distribution, with viable cell population (Annexin V^-^/PI^-^) distributed in the left lower quadrant region, early apoptotic cell population (Annexin V^+^/PI^-^) in the right lower quadrant region, late apoptotic cell populations (Annexin V^+^/PI^+^) in the right upper quadrant region, and the necrotic cell population (Annexin V^-^/ PI^+^) in the upper left quadrant region. In comparison to the control group, SPS-stimulus group showed increased apoptotic cell populations in dorsal raphe nucleus cells. The highest increase of apoptotic cells occurred in the SPS 7d group. The apoptosis rate was reported in Figure 6 (Figure 6, F = 99.322 df = 3, 20; *P* < 0.001. Tukey’s test *P* < 0.05). The rate began to decrease in the SPS 14d group. These data demonstrate that SPS induces apoptosis in dorsal raphe nucleus cells.

### Morphological change of the dorsal raphe nucleus by TEM

**Figure 7:**
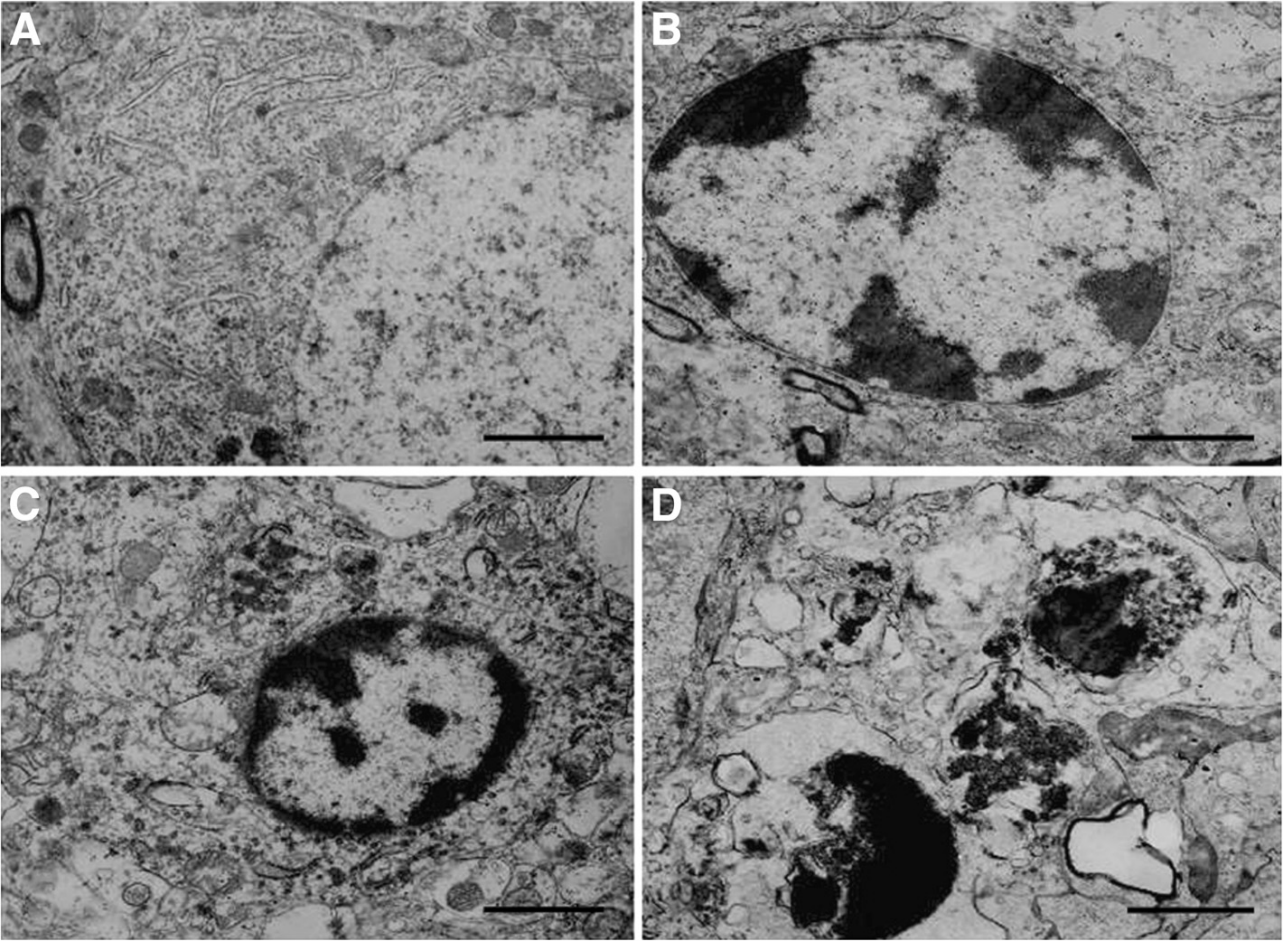
**TEM images of the dorsal raphe nucleus region. A**: control group; Dorsal raphe nucleus neuron had the nucleus is large , density of chromatin is uniform. **B**: SPS 1d group, Chromatin margination. **C**: SPS 14d group, Chromatin margination and appearance of crescent in chromatin. **D**: SPS 7d group,Appearance of apoptotic body, the cells showed some typical structural changes of apoptosis. All images are taken with identical magnification (Scale bar =1 μm ).

**Figure 8:**
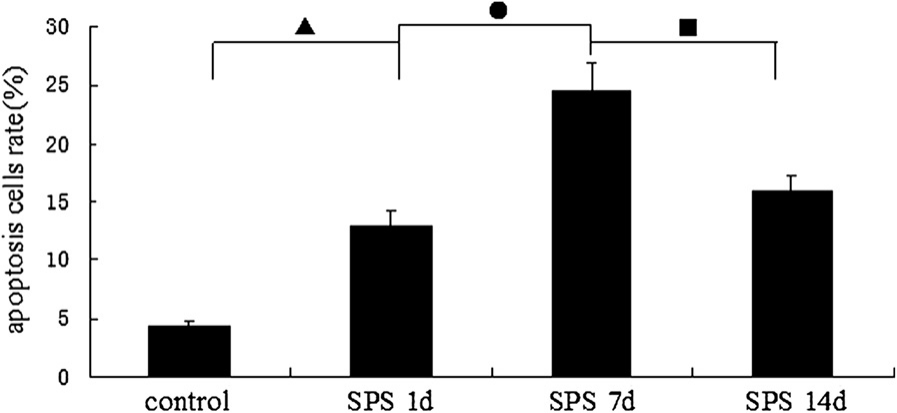
**The rates of the apoptosis cells were calculated by TEM at different time points in dorsal raphe nucleus (mean ± SEM).** The apoptosis rate peaked at SPS 7d. *P* < 0.001 SPS vs. control group. Tukey’s test: ▲*P* < 0.05 SPS 1d vs. control group. ●*P* < 0.05 SPS 7d vs. SPS 1d. ■*P* < 0.05 SPS 14d vs. SPS 7d.

As shown in Figure 7, we further performed TEM to assess the morphological changes of the dorsal raphe nucleus upon SPS stimulus. In control rats, dorsal raphe nucleus cells exhibited a normal structure (Figure 7A). However, upon SPS stimulus, the nucleus showed variable degree of structural alterations. A portion of cells in SPS stimuli rats had characteristic of apoptosis, with chromatin condensation and margination (Figure 7B,C), appearance of chromatin crescents (Figure 7C) and apoptotic body (Figure 7D). Furthermore, these changes were found mostly in group SPS 7d. Quantitative analysis of the apoptotic changes was reported in Figure 8 (Figure 8, F = 146.252 df = 3, 20; *P* < 0.001. Tukey’s test *P* < 0.05).

### Enzymohistochemistry analysis of TMP by light microscope and TEM

**Figure 9:**
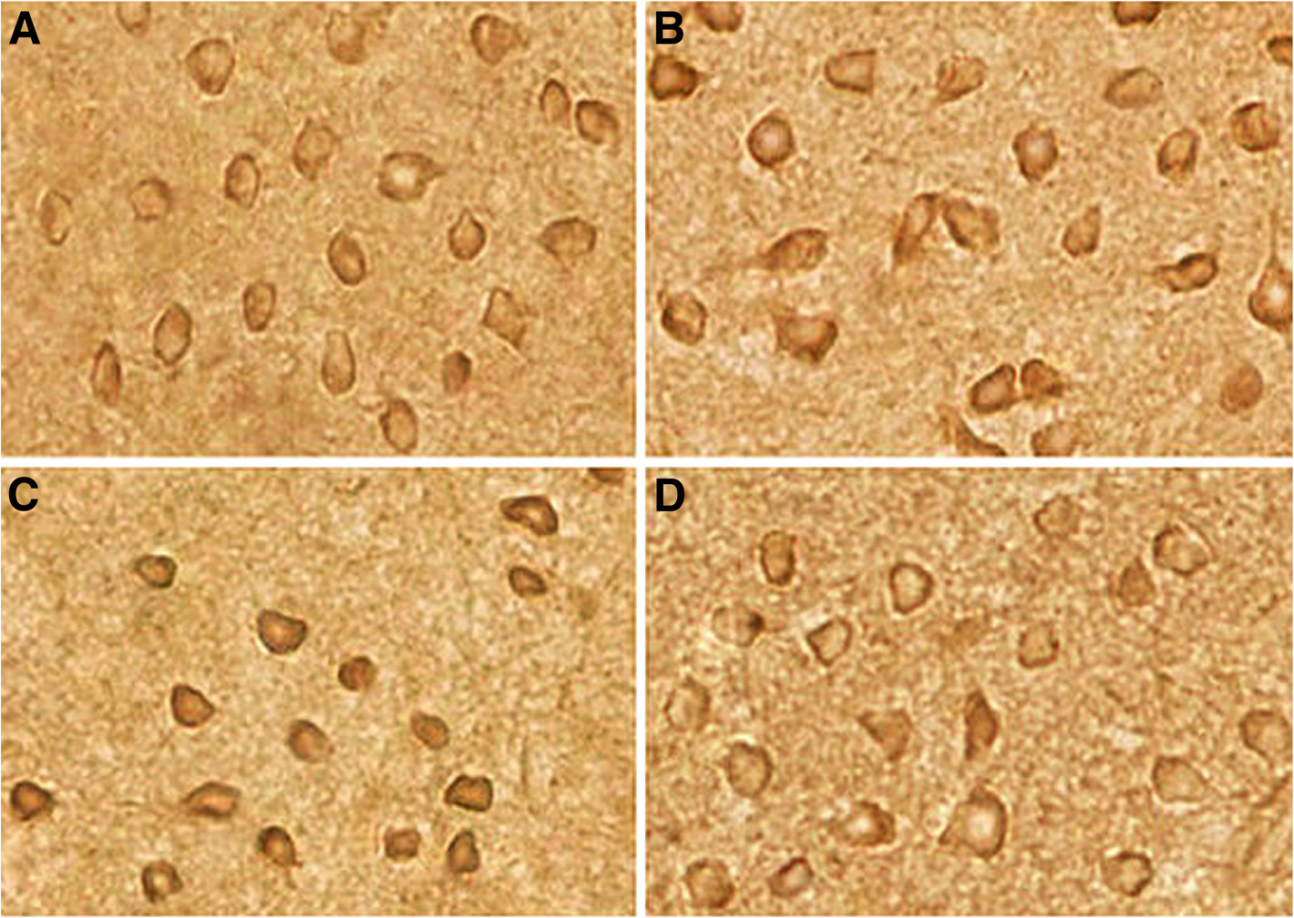
**Expression of TMP in dorsal raphe nucleus was detected by enzymohistochemistry at different time points. A**: control group; **B**: SPS1d group; **C**: SPS7d group; **D**: SPS14d group. All images are taken with identical magnification (x 400).

**Figure 10:**
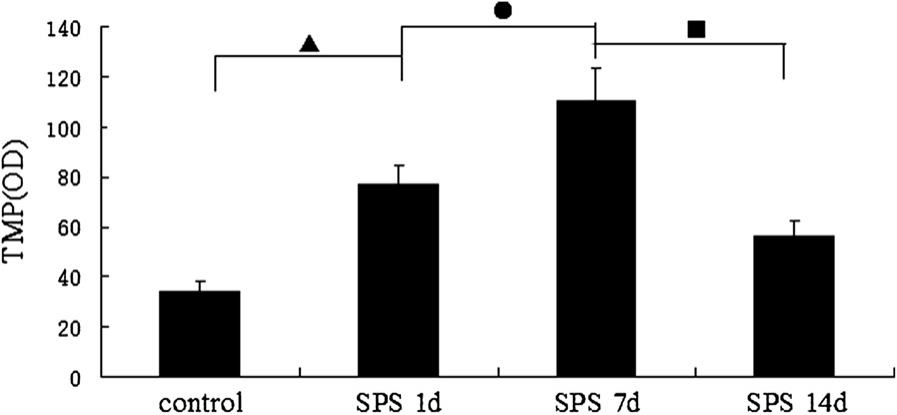
**Mean optical densities of TMP is shown at different time points in dorsal raphe nucleus dorsal raphe nucleus (mean ± SEM).** The expression of TMP peaked at SPS 7d, and then decreased gradually. *P* < 0.001 SPS vs. control group. Tukey’s test: ▲*P* < 0.05 SPS 1d vs. control group. ●*P* < 0.05 SPS 7d vs. SPS 1d. ■*P* < 0.05 SPS 14d vs. SPS 7d.

**Figure 11:**
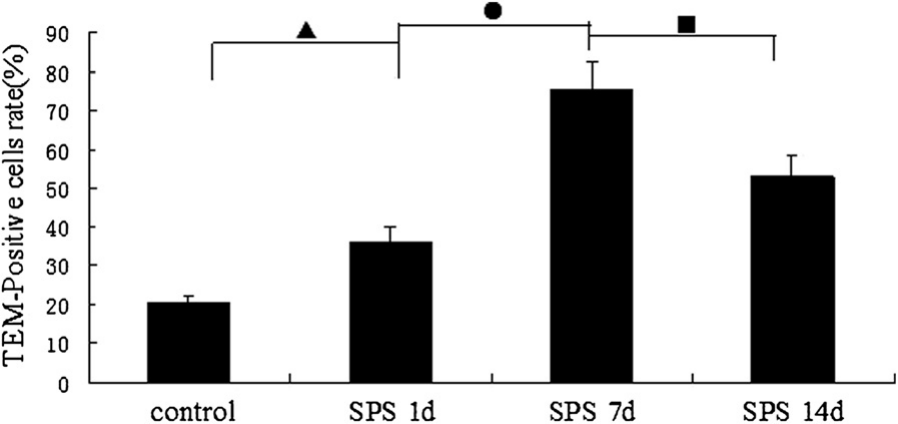
**The rate of positive cells of TMP at different time points in dorsal raphe nucleus (mean ± SEM). **The rate of positive cells peaked at SPS 7d. *P* < 0.001 SPS vs. control group. Tukey’s test: ▲*P* < 0.05 SPS 1d vs. control group. ●*P* < 0.05 SPS 7d vs. SPS 1d. ■*P* < 0.05 SPS 14d vs. SPS 7d.

We examined the distribution of thiamine monophosphatase (TMP) in samples from SPS- stimulus or control rats. Enzymohistochemistry staining analysis showed that TMP were widely distributed throughout the dorsal raphe nucleus neuron region, mainly in the cytoplasm (TMP-positive cells were stained in brown, Figure 9). There was a significant increase of TMP content in the SPS groups compared to the control group (Figure 9A-D). The increase in TMP expression peaked in group SPS 7d, and TMP expression began decrease in group SPS 14d. The mean optical densities of TMP is shown in Figure 10 (Figure 10, F = 79.623 df =3, 20; *P* < 0.001. Tukey’s test *P* < 0.05). The rate of positive cells of TMP was reported in Figure 11 (Figure 11, F = 119.084 df = 3, 20; *P* < 0.001. Tukey’s test *P* < 0.05).

**Figure 12:**
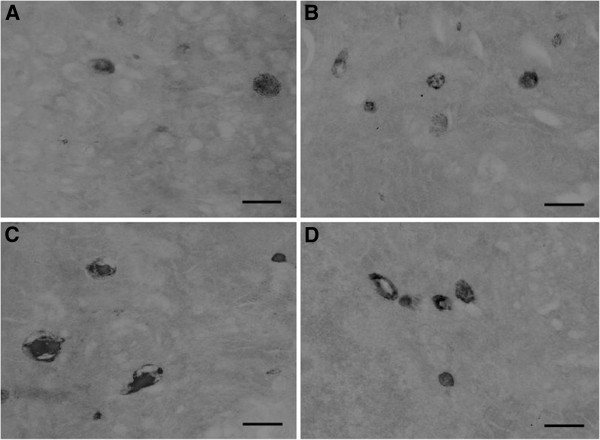
**The distribution of TMP in lysosome were found in dorsal raphe nucleus by TEM in each group, Positive expression of TMP produces high electron-dense black granule(↑). A**: control group; **B**: SPS1d group; **C**: SPS7d group; **D**: SPS14d group. The membrane disruption of lysosomes and the translocation of TMP from lysosome to cytoplasm appeared in SPS group. All images are taken with identical magnification (Scale bar =500 nm).

**Figure 13:**
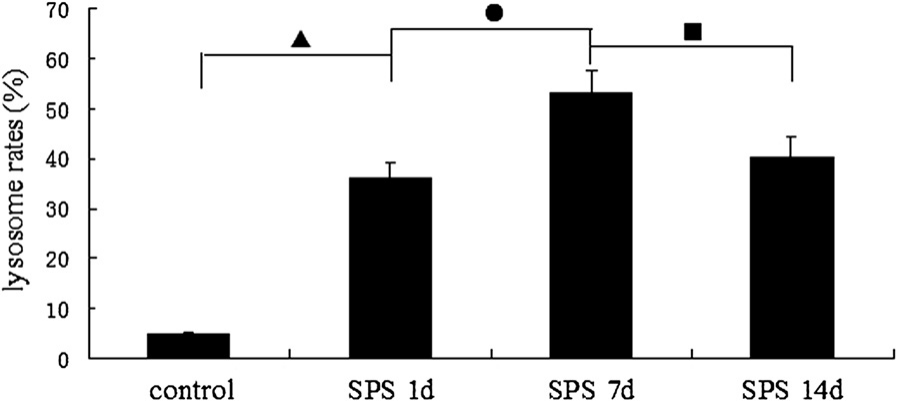
**The rates of lysosome which have positive expression of TMP were calculated by TEM at different time points in dorsal raphe nucleus (mean ± SEM).** This change was found mostly at SPS 7d. *P* < 0.001 SPS vs. control group. Tukey’s test: ▲*P* < 0.05 SPS 1d vs. control group. ●*P* < 0.05 SPS 7d vs. SPS 1d. ■*P* < 0.05 SPS 14d vs. SPS 7d.

Levels of TMP expression were also evaluated using TEM method. As shown in the Figure 12, positive TMP expression results in high electron-dense black granules that were located in the lysosomes in the dorsal raphe nucleus neuron (Figure 12A-D). Intracellular lysosomes had a normal round-shape structure in control group (Figure 12A). However, many lysosomes with abnormal shape were observed in SPS groups (Figure 12B,D). The membrane disruption of lysosomes and the translocation of TMP from lysosome to cytoplasm were also observed in SPS groups(Figure 12B,C). The numbers of lysosomes with positive expression of TMP were significantly increased in the SPS groups compared to the control group. Again, the most dramatic change occurred in group SPS 7d. The rates of lysosome which have positive expression of TMP was reported in Figure 13 (Figure 13,F = 187.044 df = 3, 20; *P* < 0.001. Tukey’s test *P* < 0.05).

## Discussion

PTSD is a disfunction in response to fear-related stimuli. Four major types of characteristic symptoms associated with PTSD are: re-experiencing, avoidance, numbing and hyperarousal [1]. Single-prolonged stress (SPS), a well established animal model for studying PTSD [26], induces enhanced inhibition of the hypothalamic-pituitary-adrenal (HPA) axis, which has been found in patients with PTSD. SPS-stimulus rats also exhibit behavioral abnormalities (enhanced anxiety) that mimic the symptoms associated with PTSD patients [19,21,27]. The dorsal raphe nucleus involves in many physiological activities, and its function has been associated to PTSD. Several lines of evidence have implicated the dorsal raphe nucleus as a main substrate for modulation of nocuous stress. Volumetric image analysis of the brainstem showed smaller gray matter volume in the pons with PTSD [7], which might be related to apoptosis. Thus, this study focused on the changes of the dorsal raphe nucleus in rat after SPS stimulus, and determined whether apoptosis occurs in the dorsal raphe nucleus after exposure to SPS.

Apoptosis is a genetically controlled, complex process that is central to normal development, homeostasis and disease development. Apoptosis is activated in response to environmental signals or being triggered by intrinsic factors. The process is designed to kill and remove errant or aging cells [28]. Apoptosis of neurons involves many cellular and molecular processes. In this study, we determined the apoptosis rate in the dorsal raphe nucleus using Annexin V-FITC/PI double-labeled flow cytometry (FCM). The results showed that SPS stimuli induced apoptosis of dorsal raphe nucleus neuron and the rate of apoptosis peaked at day 7 after SPS stimuli. We observed morphologic changes characteristic of apoptosis in the dorsal raphe nucleus neuron [29], such as chromatin condensation, chromatin margination, chromatin crescents, nucleus fragmentation, nucleus disappearance and apoptotic body.

Mitochondria play a central role in cellular survival and apoptotic death [30,31]. Mitochondria participate in apoptosis by transduction or magnification of apoptotic signal. Cyt-C, a key protein in electron transport, is located on mitochondrial inner and outer membrane [32], and also plays a key role in apoptosis induced by the mitochondrial pathway [33]. Our study indicates that Cyt-C were released from mitochondria to cytoplasm in dorsal raphe nucleus neurons after exposure to SPS. In cytoplasm, released of Cyt-C was appear in SPS 1d group and peaked in SPS 7d group. Numbers of apoptotic cells in dorsal raphe nucleus peaked at SPS 7d, determined by Annexin V-FITC/ PI double-labeled FCM analysis. These results demonstrated that the release of Cyt-C occured earlier than that of the apoptotic peak. These results support the notion that the mitochondrial signaling pathway is a critical process of apoptosis in dorsal raphe nucleus in the rat model of PTSD. We also examined the effect of SPS on activity of Cyt-C by Western blotting and demonstrated that the levels of Cyt-C increased upon SPS treatment.

Several signals can trigger apoptosis via different pathways, involving a variety of enzymatic reactions, including proteolytic enzymes from lysosome. TMP protease is the hallmark enzyme of lysosome, and its activity represents the status of lysosomal function. Sheen et al. reported that apoptosis increases the permeability of lysosome and leads to the release of proteases and protein kinases [17]. In this study, we measured the TMP levels in the dorsal raphe nucleus using enzymohistochemistry. We found that TMP activity was upregulated and TMP enzymes were released from lysosome upon SPS stimuli, with the highest change occurred in SPS 7d group. Thus, SPS leads to the dysfunction of lysosome of dorsal raphe nucleus, increase of permeability and rupture of lysosomal membrane. This further leads to TMP released from lysosome to cytoplasm. Increased TMP in cytoplasm facilitates the degradation, dispose and clearance of apoptotic cells products. Thus, apoptosis and overexpression of TMP may be one of the pathophysiological foundation for the behavior disorder in PTSD.

## Conclusion

Our results indicate that SPS induces Cyt-C release, apoptosis, and increased TMP in dorsal raphe nucleus neuron in rats. This may result in reduced volume of the dorsal raphe nucleus. This may present a possible mechanism that leads to reduced volume of pons and gray matter associated with PTSD.

## Abbreviations

ACTH: Adrenocorticotropic hormone; ANOVA: Analysis of variance; Cyt-C: Cytochrome c; ECL: Enhanced chemiluminescence; HPA: Hypothalamic-pituitary-adrenal; OD: Optical density; PAGE: Polyacrylamide gel electrophoresis; PBS: Phosphate-buffered saline; P FA: Paraformaldehyde; PTSD: Post-traumatic stress disorder; PVDF: Polyvinylidene fluoride; SDS: Sodium dodecyl sulfate; SPS: Single-prolonged stress; TBS: Tris-buffered saline; TBST: Tris buffered saline tween; TEM: Transmission electron microscopy; TMP: Thiamine monophosphatase.

## Competing interests

The authors declare that they have no competing interests.

## Authors’ contributions

Dongjuan Liu conceived of the study and drafted the manuscript, all authors participated in the design and coordination of the study, and Bing Xiao performed the statistical analysis. All authors read and approved the final manuscript.

## Pre-publication history

The pre-publication history for this paper can be accessed here:

http://www.biomedcentral.com/1471-244X/12/211/prepub
